# Salivary and urinary microRNAs as non-invasive biomarkers of minimal residual disease in pediatric acute lymphoblastic leukemia

**DOI:** 10.17305/bb.2026.13119

**Published:** 2026-02-19

**Authors:** Alejandra Pando-Caciano, Victoria Godoy-Vila, Sergio Murillo-Vizcarra, Monica J Pajuelo, Holger Maita-Malpartida

**Affiliations:** 1 Departamento de Ciencias Celulares y Moleculares, Facultad de Ciencias e Ingeniería, Universidad Peruana Cayetano Heredia, Lima, Peru; 2 Sub-Unit of Research and Technological Innovation, Instituto Nacional de Salud del Niño San Borja, Lima, Peru; 3 Hematopoietic Stem Cell Transplantation Service, Instituto Nacional de Salud del Niño San Borja, Lima, Peru; 4 Specialized Hematology and Hematopoietic Stem Cell Transplantation Service, Hospital Nacional Edgardo Rebagliati Martins, Lima, Peru

**Keywords:** Child, leukemia, lymphoblastic, acute, minimal residual disease, micrornas, biomarkers, noninvasive diagnostic techniques

## Abstract

The measurement of minimal residual disease (MRD) in bone marrow serves as a significant predictor of relapse in acute lymphoblastic leukemia (ALL). MicroRNAs, which are secreted in urine and saliva, typically reflect the cellular state and function, thereby demonstrating potential as non-invasive biomarkers for predicting MRD status. This study aims to evaluate the utility of circulating microRNAs in saliva and urine as MRD biomarkers in pediatric patients with ALL. The study cohort comprised 106 patients under 18 years of age diagnosed with ALL at a national pediatric referral hospital in Lima, Peru. Saliva and urine samples were collected on day 15 of induction chemotherapy. Patients were categorized into high-risk relapse (HRR) and standard/intermediate-risk relapse (SIRR) groups based on their day-15 MRD status. Small RNA sequencing was conducted on six paired samples, while RT-qPCR was performed on 23 paired samples to identify and validate differentially expressed microRNAs. A total of thirty microRNAs were differentially expressed in saliva, and two in urine, both of which were downregulated. In saliva, 22 microRNAs showed significant upregulation. miR-1246, miR-223-3p, and miR-1290 exhibited the highest levels of upregulation in HRR patients (log_2_ fold change = 4.00, 3.95, and 3.73, respectively). Validation confirmed the upregulation of miR-223-3p (log_2_ fold change = 1.15, *P ═* 0.034). miR-223-3p, both alone and in combination with miR-1290 and miR-1246, demonstrated optimal performance in distinguishing HRR from SIRR patients (AUC = 0.68, 95% CI: 0.52–0.84; AUC = 0.69, 95% CI: 0.53–0.84, respectively). These preliminary findings indicate that miR-223-3p, whether used independently or in conjunction with miR-1246 and miR-1290, may serve as promising non-invasive MRD biomarkers in pediatric ALL. Further validation in larger patient cohorts is necessary to corroborate these results.

## Introduction

Acute lymphoblastic leukemia (ALL) is the most prevalent type of childhood cancer globally, with an incidence of 34 cases per million [1]. Significant advancements in treatment modalities have elevated the 5-year survival rate to approximately 90% [2]. However, despite these advances, around 15%–20% of patients experience relapse [3].

The risk of relapse is evaluated through various modalities, with minimal residual disease (MRD) measured at multiple time points during therapy serving as a critical prognostic marker [4]. MRD assessment is conducted using flow cytometry, quantitative polymerase chain reaction (qPCR), and Next Generation Sequencing (NGS) approaches on bone marrow (BM) samples [5]. BM aspiration is recognized as the gold standard for MRD assessment due to its significantly higher sensitivity compared to peripheral blood [6]. Nonetheless, the collection of BM samples in pediatric patients is often hindered by the pain and anxiety experienced by the children and their families [7].

MicroRNAs (miRNAs) are short (∼22 nucleotides) non-coding RNA sequences that primarily regulate gene expression at the post-transcriptional level, as well as through transcriptional repression [8, 9]. MiRNAs can be released into various extracellular fluids, including non-invasive specimens such as saliva and urine [10]. Previous studies have shown that cancer patients release higher levels of specific miRNAs compared to healthy individuals, and that these levels correlate with disease progression—being more pronounced in advanced stages than in early stages—indicating that miRNAs may play a vital role in cancer development and progression [11]. These findings suggest that miRNAs could function as oncogenes or tumor suppressor genes by silencing the expression of tumor suppressor genes and oncogenes, respectively [12].

In pediatric ALL, various circulating miRNAs (e.g., miR-31, miR-128-3p, miR-107, miR-155, miR-124, miR-181, miR-100, miR-223, miR-103a) have been identified in serum and plasma samples and evaluated as potential biomarkers for disease diagnosis and MRD monitoring, demonstrating high sensitivity and specificity [13–20]. While dysregulated expression of specific miRNAs has been observed in the saliva and urine of patients with solid tumors, their potential as non-invasive MRD biomarkers in pediatric ALL remains unexplored [21–23]. This study aims to evaluate the utility of circulating miRNAs in saliva and urine as non-invasive MRD biomarkers in pediatric ALL, providing a significant advantage over current MRD monitoring practices due to its non-invasive nature and ease of sampling.

## Materials and methods

### Study design and patients

This study was conducted in two phases: a discovery phase and a validation phase. Small RNA sequencing was performed to identify differentially expressed miRNAs during the discovery phase. The validation phase consisted of a technical validation phase and a clinical validation phase. Differentially expressed miRNAs were validated by quantitative reverse transcription polymerase chain reaction (RT-qPCR) during the technical validation phase using the same samples analyzed in the Small RNA sequencing. The discrimination accuracy of the validated miRNAs was subsequently assessed in a partially independent group of patients during the clinical validation phase.

The study population comprised children under 18 years diagnosed with ALL by flow cytometry on BM, who were treated at the Instituto Nacional de Salud del Niño San Borja (INSN-SB) in Lima, Peru.

### Sample and data collection

Samples were collected on day 15 of induction chemotherapy. Depending on the patient’s age, urine was collected using a sterile plastic collection bag or container, while saliva was collected using the Salivette^®^ system (Sarstedt, Nümbrecht, Germany) according to the manufacturer’s instructions.

Both urine and saliva samples were collected as the first specimen of the day, prior to any food or drink consumption, to minimize variability associated with oral intake. Immediately after collection, samples were centrifuged at 4,800× g for 15 min at 4 ^∘^C to eliminate cellular debris and food residues. The supernatants were aliquoted (350 µL) and stored at --80 ^∘^C until miRNA isolation.

Clinical and sociodemographic data—including age, sex, city of origin, white blood cell count, platelet count, neutrophil count at diagnosis, prednisone response on day 8 of induction, BM MRD on day 15, and molecular and cytogenetic profiles—were extracted from patients’ electronic medical records. Based on the MRD at day 15, patients were classified as high-risk relapse (HRR) (MRD ≥ 10%) or Standard Risk Relapse (SIRR) (MRD < 10%) according to the ALL IC-BFM 2009 protocol [24]. BM MRD at day 15 was assessed by flow cytometry.

### miRNA isolation, quantification, and purity assessment

miRNAs were isolated using the mirVana™ miRNA Isolation Kit (Invitrogen™ , Waltham, MA, USA) from 350 µL of supernatant, following the manufacturer’s protocol. Following the denaturation step, 10 µL of synthetic cel-miR-39 miRNA mimic (5′ UCACCGGGUGUAAAUCAGCUUG 3′) at a concentration of 571.43 pmol/L was added to each sample to monitor the miRNA isolation process.

Total RNA concentration was measured using a Qubit 4.0 fluorometer (Invitrogen™ , Waltham, MA, USA) with the Qubit™ RNA HS (High Sensitivity) Assay Kit (Invitrogen™, Waltham, MA, USA). RNA purity was assessed with a Nanodrop 2000 spectrophotometer (Thermo Fisher Scientific, Waltham, MA, USA) by analyzing the 260/280 absorbance ratio. Samples with a 260/280 ratio of approximately 2.0 were considered pure.

### Small RNA Sequencing

Small RNA libraries were prepared using the QIAseq miRNA Library Kit (Qiagen, Hilden, Germany), in accordance with the manufacturer’s instructions. The libraries were then pooled and sequenced on a NovaSeq 6000 instrument (Illumina Inc., San Diego, California, USA) with 75 bp single-end reads.

Raw reads were processed with Reaper to remove 3′ adapter sequences and assess read quality [25]. Trimmed reads were aligned to mature miRNAs downloaded from miRbase (v22.1) using Bowtie 2.0 (v 2.5.1) in end-to-end mode, allowing a maximum of one mismatch per read.

Differential expression analysis was conducted using the DESeq2 package in R (version 4.3.1; R Core Team, Vienna, Austria), within the RStudio environment (Posit Software, Boston, MA, USA) [26]. miRNAs were classified as differentially expressed when the adjusted *P* value was below 0.05. Heatmaps and volcano plots were utilized to visualize differentially expressed miRNAs (DEMs). To identify outlier samples and select preliminary candidates for RT-qPCR validation, read counts from the most upregulated and downregulated miRNAs were examined using count plots. Final candidates for validation were selected based on previous associations with leukemogenesis.

### Selection of reference miRNAs for RT-qPCR data normalization

Data obtained from sequencing underwent three initial filtering steps. The first step involved selecting miRNAs with an adjusted *P* value greater than 0.05. Subsequently, miRNAs with log_2_ fold change (FC) values less than --0.1 or greater than 0.1 were excluded. Finally, miRNAs with average read counts below 500 were removed to ensure an adequate number of sequences for detection by RT-qPCR.

The filtered sequences were input into the online software RefFinder to identify the most stable miRNAs [27]. The three most stable miRNAs, not previously associated with cancer development, along with two additional candidates suggested by a prior study (miR-16-5p and miR-25), were amplified by RT-qPCR [28]. The qPCR data were subsequently analyzed for stability using geNorm to determine the most stable miRNAs. geNorm calculates the gene expression stability value (M) by pairwise comparison of a specific gene with all other candidate genes, with the gene exhibiting the lowest M value considered the most stable [29].

### cDNA synthesis and qPCR

cDNA was synthesized from 10 ng of total RNA using the TaqMan™ Advanced miRNA cDNA Synthesis Kit (Applied Biosystems™ , Waltham, MA, USA), which includes poly(A) tailing, adaptor ligation, reverse transcription (RT), and a pre-amplification step (miR-Amp) according to the manufacturer’s protocol. The synthesized cDNA was stored at --20 ^∘^C until qPCR analysis.

miRNA expression was quantified using individual TaqMan™ Advanced miRNA Assays (Thermo Scientific, Waltham, MA, USA), following the manufacturer’s instructions. The assays utilized in this study are listed in Table S1. Each sample was analyzed in triplicate on a LightCycler^®^ 480 System (Roche, Mannheim, Germany) under the following thermal cycling conditions: 20 s at 95^∘^C followed by 40 cycles of 1 second at 95^∘^C and 20 s at 60^∘^C. Results from qPCR were analyzed using GenEx™ software (MultiD Analyses AB, Gothenburg, Sweden). The geometric mean of the most stable miRNAs was used for normalization, and fold change was calculated using the 2 - ΔΔCq method [30].

Samples were excluded from downstream analysis if the exogenous spike-in *(cel-miR-*39) showed no amplification or Cq values greater than 35 cycles.

### Ethical statement

Written informed consent was obtained from one parent to permit their children’s participation in the study. The Institutional Ethics Committee of the INSN-SB (Protocol No. 627-21) and the Universidad Peruana Cayetano Heredia (Protocol No. 206958) approved the study.

### Statistical analysis

The expression levels of miRNAs between the two risk groups were compared using Wilcoxon’s test. ΔCq values, defined as ΔCq = Cq_miRNA_ - Cq_GM(ref miRNAs)_, served as predictor variables. Both univariate and multivariate logistic regression analyses, as well as receiver operating characteristic (ROC) curves, were employed to evaluate the capacity of individual miRNAs and the combined 3-miRNA panel (miR-1290, miR-223-3p, and miR-1246) to differentiate between the HRR and SIRR groups. In the univariate models, optimal cut-off values were derived directly from the ΔCq distributions. For the multivariate model, the cut-off was established from the predicted probabilities of the logistic regression model, incorporating the ΔCq values of all three miRNAs. The area under the ROC curve (AUC) and corresponding 95% confidence intervals (CI) were estimated using the pROC package, and differences between AUCs were assessed using the DeLong test. The optimal cut-off, sensitivity, and specificity were defined based on the maximum Youden index. Optimism was quantified through bootstrap internal validation (1,000 resamples) for both univariate and multivariate logistic models. Corrected AUC values were calculated by subtracting the estimated optimism from the original AUC.

All statistical analyses and graphing were conducted using R (version 4.3.1; R Core Team, Vienna, Austria) within the RStudio environment (Posit Software, Boston, MA, USA). A *P* value of less than 0.05 was regarded as statistically significant.

## Results

### Patient characteristics

Saliva and urine samples were collected from 106 patients. In the discovery phase, six patients from each risk group, matched by age and sex, were included in the study (*n* ═ 12). Among the remaining 94 patients, 34 were classified into the HRR group. Of these, only 23 had at least one saliva sample available for RNA isolation, primarily due to challenges in sample collection from infants. These 23 patients, along with their age and sex-matched counterparts from the SIRR group and three additional pairs analyzed in the discovery phase with available RNA samples, were included in the validation phase. Three patients in the HRR group were excluded due to the absence of amplification in the internal control (*cel-miR-39*) (Table S2), resulting in a total of 23 patients from the HRR group and 26 patients from the SIRR group analyzed during the validation phase (*n* ═ 49).

Table 1 summarizes the key sociodemographic, clinical, and hematological characteristics of the patients included in the study. The median MRD levels for the HRR group were 54.68% in the discovery phase and 26.90% in the validation phase, while the MRD levels for the SIRR group were 1.26% in the discovery phase and 1.27% in the validation phase.

**Table 1:** Sociodemographic, clinical, and hematological characteristics of study participants

	**Discovery phase** **(*n* ═ 12)**	**Validation phase (*n* ═ 49)**
**Variable**	***n* (%)**	
Sex		
Female Male	6 (50) 6 (50)	12 (24.49) 37 (75.51)
City of birth		
Lima Other regions	8 (66.67) 4 (33.33)	27 (55.10) 22 (44.90)
Cytogenetic abnormalities		
Structural and numerical abnormalities t(2;19)(p13;p13) +5 del(6)(q13q23)inv(9)(p12q13)add(11)(q13) [17]t(9;22)(q34;q11.2)Complex karyotype (≥3 abnormalities)Normal karyotypeUnavailable results	1 (8.33) 1 (8.33) 0 (0) 0 (0) 0 (0) 0 (0) 0 (0) 2 (16.67) 4 (33.33) 5 (41.67)	6 (12.24) 1 (2.04) 1 (2.04) 1 (2.04) 1 (2.04) 1 (2.04) 1 (2.04) 8 (16.33) 20 (43.48) 15 (30.61)
Genetic abnormalities		
BCR::ABL p190 BCR::ABL p210 ETV6::RUNX1 TCF3::PBX1 KMT2A::AFF1 None of the above Unavailable results	0 (0) 0 (0) 1 (8.33) 0 (0) 0 (0) 10 (83.33) 1 (8.33)	1 (2.04) 1 (2.04) 4 (8.16) 4 (8.16) 0 (0) 39 (79.59) 0 (0)
Immunophenotype		
B T	12 (100) 0 (0)	43 (87.76) 6 (12.24)
Day 8 prednisone response		
Good (<1,000 blasts/ µL) Poor (≥ 1,000 blasts/ µL) Unavailable results	9 (75) 3 (25) 0 (0)	36 (73.47) 12 (24.49) 1 (2.04)
	**Median (IQR)**	
Age (years)	7.67 (6.99)	8.01 (5.48)
Bone marrow blasts (%)	76.04 (24.63)	79 (28.46)
White blood cells (10^3^ cells/µL)	23.84 (63.86)	28.90 (98.83)
Neutrophils (10^3^ cells/µL)	2.13 (5.11)	1.48 (4.33)
Platelets (10^3^ cells/µL)	77.50 (64)	48 (66)
Hemoglobin (g/dL)	7.75 (3.93)	8.40 (4.20)
Day 15 MRD (%)		
HRR SIRR	54.68 (60.07) 1.26 (2.70)	26.90 (37.47) 1.27 (3.64)

Abbreviations: MRD: Minimal residual disease; HRR: High-risk of relapse; SIRR: Standard intermediate-risk of relapse; IQR: Interquartile range; t: Translocation; del: Deletion; inv: Inversion.

### Discovery phase

In both saliva and urine libraries, base quality scores remained consistently high across read positions (median ≥ Q30), with no evidence of deterioration toward the 3′ end of the reads. The read length distribution showed the expected enrichment within the 21–24 nt range, consistent with canonical miRNA sizes.

**Figure 1.**
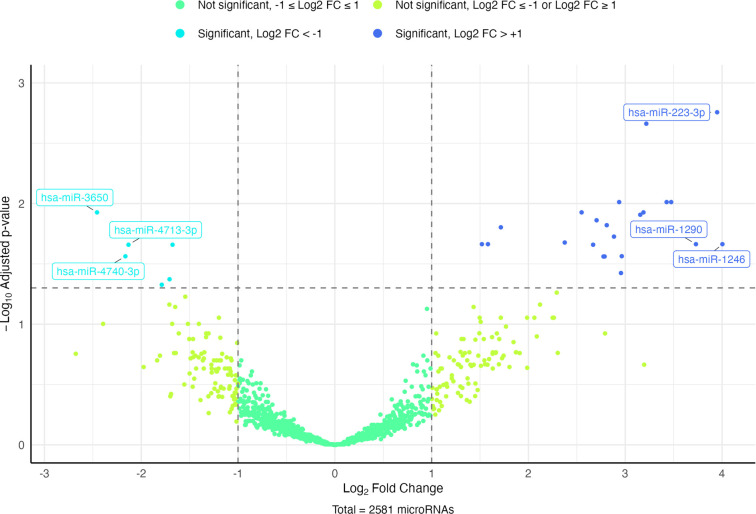
**Volcano plot of differentially expressed salivary miRNAs in the discovery phase.** The plot shows differential miRNA expression between relapse-risk groups (HRR vs SIRR) measured by small RNA sequencing. Each point represents one detected miRNA (total = 2,581), with the x-axis indicating log_2_ fold change (HRR vs SIRR) and the y-axis indicating −log_10_ adjusted *P* value. Of the 2,581 detected miRNAs, 1,541 had valid adjusted *P* values and are displayed in the volcano plot; 1,040 miRNAs had adjusted *P* values equal to NA and therefore cannot be represented when plotting −log_10_(padj). Points are color-coded by direction of change and statistical significance based on adjusted *P* values, and the horizontal dashed line denotes the significance threshold (adjusted *P ═* 0.05). The three most upregulated miRNAs (miR-223-3p, miR-1290, miR-1246) and the three most downregulated miRNAs (miR-3650, miR-4713-3p, miR-4740-3p) are highlighted. Abbreviations: HRR: High-risk of relapse; SIRR: Standard/intermediate-risk of relapse.

Small RNA sequencing analysis identified 32 differentially expressed miRNAs between the HRR and SIRR groups, with 30 miRNAs identified in saliva and 2 in urine (Figure 1, Tables S3 and S4). In saliva, 22 miRNAs were upregulated, and 8 were downregulated, whereas both differentially expressed miRNAs in urine were downregulated (Tables S3 and S4). Notably, miR-1290, miR-223-3p, and miR-1246 exhibited the highest upregulation, while miR-3650, miR-4740-3p, and miR-4713-3p demonstrated the most significant downregulation in saliva (Figure 1 and Table S3).

Following a significant increase in mean counts in at least 50% of the samples in the HRR group compared to the SIRR group, and confirming the previous association of the most upregulated miRNAs in saliva with leukemogenesis, only these miRNAs (miR-1290, miR-1246, and miR-223-3p) were selected for further validation by RT-qPCR (Figure S1). The downregulated miRNAs in saliva and urine were excluded from subsequent analysis due to a lack of distinguishable differences in mean counts between the HRR and SIRR groups (Figures S2 and S3). Additionally, no previous association with leukemogenesis could be confirmed.

### Reference miRNAs for RT-qPCR data normalization

The most stable miRNAs identified from the sequencing data were miR-6749-5p, miR-12121, and miR-6722-3p. Notably, these miRNAs were not detected by RT-qPCR. However, consistent with previous literature, amplification was observed for miR-16-5p and miR-25. Based on the geNorm stability value (M), miR-16-5p and miR-25 were determined to be the most stable miRNAs (Table S5). Therefore, the geometric mean of their Cq values was used to normalize the expression levels of the miRNAs during the validation phase.

### Technical validation phase

During the technical validation phase, elevated expression levels of all three miRNAs in the HRR group compared to the SIRR group were confirmed, consistent with the discovery phase findings. Specifically, the log_2_ FC for miR-1290, miR-1246, and miR-223-3p was 0.12 (FC = 1.09), 0.25 (FC = 1.19), and 2.80 (FC = 6.98), respectively, which were notably lower than those obtained during the initial phase of the study.

### Clinical validation phase

**Figure 2.**
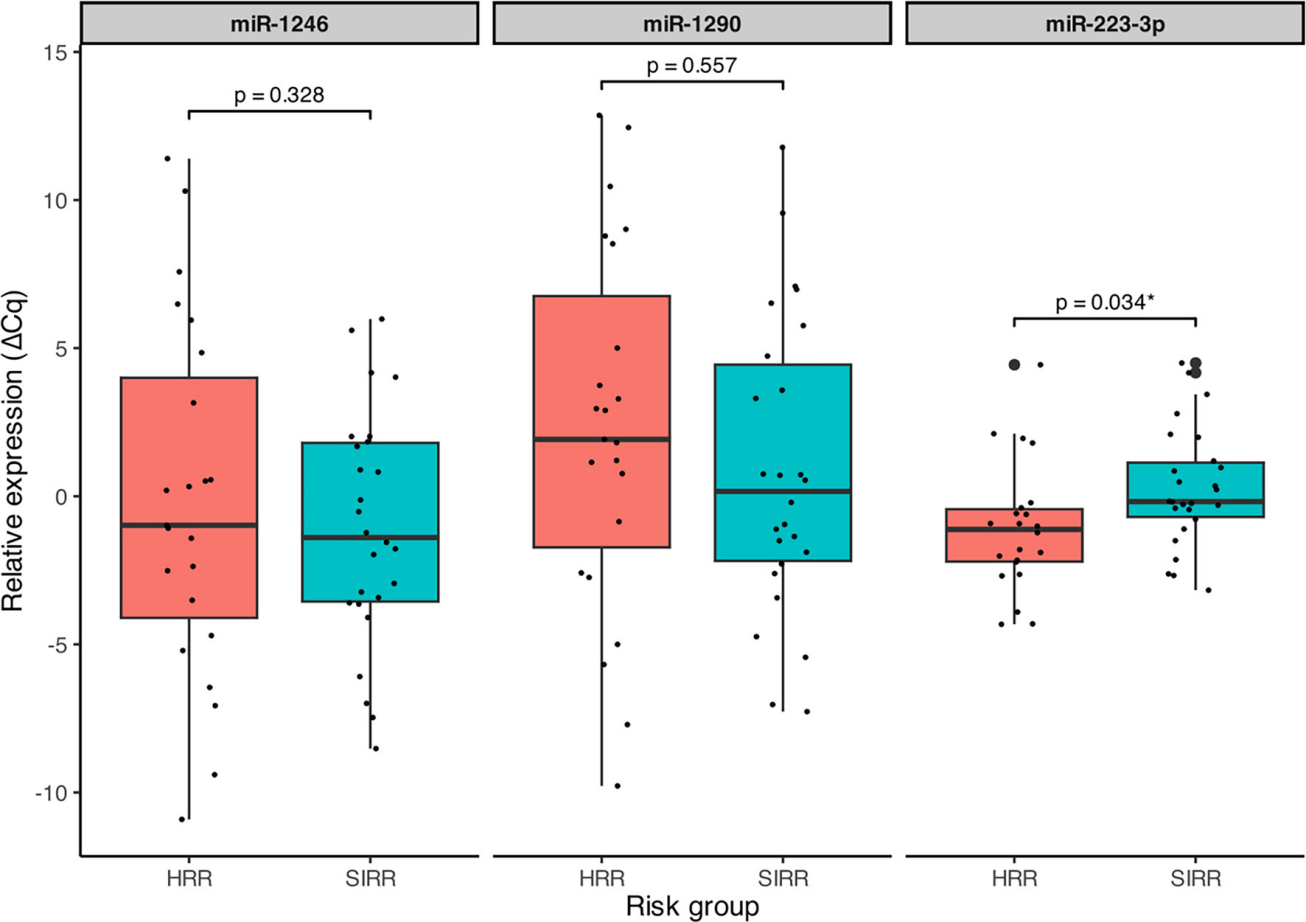
**Relative expression of miR-1246, miR-1290, and miR-223-3p by relapse-risk group in the clinical validation cohort.** Salivary miRNA expression was quantified by RT-qPCR and summarized as ΔCq values normalized to the geometric mean of the reference microRNAs (miR-16-5p and miR-25). Lower ΔCq values indicate higher relative miRNA expression. Boxplots display the median and interquartile range, with whiskers representing the data spread and individual samples overlaid as points. Between-group differences were evaluated using Wilcoxon’s test, with *P* values shown above brackets; the asterisk denotes statistical significance (*P* < 0.05). Abbreviations: HRR: High-risk of relapse; RT-qPCR: Reverse transcription quantitative polymerase chain reaction; SIRR: Standard/intermediate-risk of relapse; ΔCq: Delta quantification cycle (ΔCq = Cq miRNA - Cq geometric mean of reference miRNAs).

The mean values for ΔCq, FC, and *P* values for each miRNA are presented in Table 2. A statistically significant difference in expression levels of miR-223-3p between the risk groups was observed, with this miRNA demonstrating greater than twofold upregulation in the high-risk group compared to the standard-intermediate risk group (FC = 2.22), as illustrated in Figure 2.

**Figure 3.**
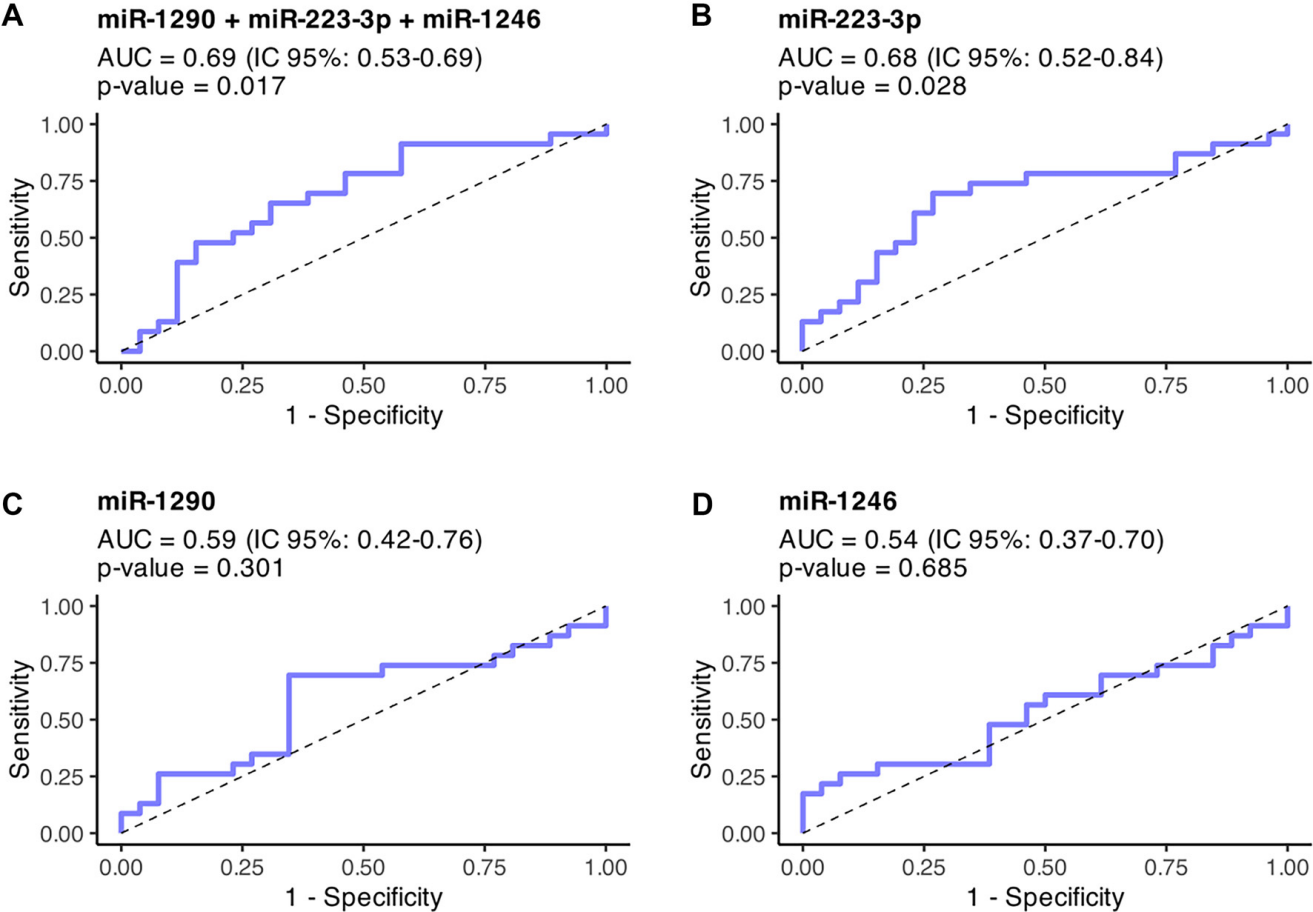
**ROC curves for discrimination of relapse-risk groups using individual salivary miRNAs and a three-miRNA panel.** ROC curves depict the ability of (A) the combined three-miRNA panel (miR-1290, miR-223-3p, miR-1246) and the individual markers (B) miR-223-3p, (C) miR-1290, and (D) miR-1246 to distinguish HRR from SIRR patients in the clinical validation cohort. The three-miRNA curve was derived from a multivariable logistic regression model, whereas single-marker curves were derived from univariate logistic regression models. The AUC, 95% confidence interval, and model *P* value are shown within each panel. The diagonal dashed line indicates no-discrimination performance. Comparison of AUCs showed no significant difference between the three-miRNA panel and miR-223-3p alone (DeLong test, *P ═* 0.889). Abbreviations: AUC: Area under the curve; CI: Confidence interval; HRR: High-risk of relapse; ROC: Receiver operating characteristic; SIRR: Standard/intermediate-risk of relapse.

ROC curve analysis indicated that miR-223-3p, both alone and in combination with miR-1290 and miR-1246, was capable of distinguishing between the HRR and SIRR patient groups, yielding AUC values of 0.68 (95% CI: 0.52–0.84, *P* value = 0.028) and 0.69 (95% CI: 0.53–0.84, *P* value = 0.017) respectively (Figure 3). Notably, combining the three miRNAs did not significantly enhance the individual discriminatory capacity of miR-223-3p (DeLong test, *P ═* 0.889).

Table 3 presents the sensitivity, specificity, and cut-off value for each miRNA. The highest sensitivity and specificity were noted for miR-223-3p, with a cut-off value of --0.51. Table 3 also includes the AUC corrected for optimism, which showed only minimal deviation from the original estimates (optimism values ranging from 0.000 to 0.036), suggesting that the reported performance metrics are robust. For exploratory purposes, an additional AUC analysis excluding samples from the discovery phase was performed, with results provided in Table S6.

**Table 2:** Average ΔCq, fold change, and *P* value for miR-1246, miR-1290, and miR-223-3p during the clinical validation phase

**miRNA**	**Average **Δ**Cq in the HRR group**	**Average **Δ**Cq in the SIRR group**	**Log_2_ FC**	***p* value**
miR-1290	2.28	0.96	–1.32	0.328
miR-1246	–0.19	–1.31	–1.13	0.557
miR-223-3p	–0.99	0.16	+1.15	0.034*

ΔCq is defined as the difference between the target miRNA’s Cq value and the geometric mean of reference miRNAs. The log_2_ fold change (log_2_FC) is calculated using the formula: Log_2_FC ═ --(mean ΔCq_HRR_ -- mean ΔCq_SIRR_), where HRR represents high risk of relapse and SIRR denotes standard intermediate risk of relapse. A statistically significant result is indicated by *P < 0.05. Abbreviations: HRR: High-risk of relapse; SIRR: Standard intermediate-risk of relapse; ΔCq: Delta quantification cycle (ΔCq = Cq miRNA - Cq geometric mean of reference miRNAs).

**Table 3:** ROC analysis and diagnostic performance metrics for miR-1290, miR-223-3p, miR-1246, and the combined three-miRNA panel

**miRNA**	**Original AUC**	**95% CI**	***P* value**	**Optimism**	**Corrected AUC**	**95% CI**	**Youden index**	**Sensitivity**	**95% CI**	**Specificity**	**95% CI**
3-miRNA panel	0.69	0.53-0.84	0.017	0.036	0.65	0.53-0.82	0.48	0.70	0.48-0.87	0.73	0.54-0.88
miR-1290	0.59	0.42-0.76	0.301	0.007	0.58	0.44-0.75	0.76	0.52	0.70-0.87	0.65	0.46-0.85
miR-223-3p	0.68	0.52-0.84	0.028	0.000	0.68	0.51-0.82	--0.51	0.70	0.52-0.87	0.73	0.54-0.88
miR-1246	0.54	0.37-0.70	0.685	0.031	0.50	0.41-0.68	4.51	0.26	0.09-0.48	0.92	0.81-1.00

In single-marker models, optimal cut-off values were directly derived from the ΔCq distributions. For the combined three-miRNA panel, the cut-off was established based on predicted probabilities from a multivariable logistic regression model that incorporated the ΔCq values of all three miRNAs. To estimate optimism, bootstrap internal validation was conducted with 1,000 resamples, and corrected AUC values were calculated by subtracting the estimated optimism from the original AUC. Sensitivity and specificity were assessed at the optimal cut-off determined by the Youden index. Abbreviations: ROC: Receiver operating characteristic; AUC: Area under the curve; CI: Confidence interval; ΔCq: Delta quantification cycle (ΔCq = Cq miRNA - Cq geometric mean of reference miRNAs).

## Discussion

The excretion and abnormal expression of miRNAs (miR-155, miR-21, and miR-210) in the serum of patients with diffuse large B-cell lymphoma were first reported by Lawrie *et* al. in 2008. Since then, numerous studies have investigated the diagnostic, prognostic, and treatment monitoring applications of these sequences [31].

In this study, we identified urinary and salivary miRNAs that are differentially expressed between ALL patients with HRR and those with SIRR, underscoring their potential utility as non-invasive MRD biomarkers. To our knowledge, this is the first study to explore the miRNA profile in saliva and urine samples from pediatric ALL patients. Our findings provide preliminary evidence to support future investigations aimed at assessing the diagnostic or prognostic applications of miRNAs secreted in such samples, applicable not only to this specific form of pediatric cancer but also to other related malignancies.

The three most upregulated miRNAs in saliva identified by sequencing and validated by RT-qPCR—miR-1246, miR-1290, and miR-223-3p—have previously been implicated in the pathogenesis of acute leukemias, suggesting their potential role as oncomiRs in these diseases.

Upregulation of miR-1246 has been documented in patients with relapsed leukemia and in chemoresistant leukemia cell lines [32, 33]. Inhibition of this miRNA led to a reversal of drug resistance *in vitro* by inducing cell apoptosis and blocking cell cycle progression. Additionally, increased levels of miR-1246 have been observed in exosomes derived from patients with acute myeloid leukemia (AML) and in xenograft mouse models [34, 35]. The upregulation of miR-1246 correlated with tumor burden and disease progression in AML xenografts [34]. Furthermore, miR-1246 levels were significantly higher in patients exhibiting elevated MRD (>20%) after induction therapy, suggesting its potential as a minimally invasive biomarker for monitoring treatment response. High expression of miR-1246 has also been noted in extracellular vesicles (EVs) from AML cells compared to those from normal cells [36]. Increased expression of miR-1246 using a miRNA mimic resulted in enhanced cell viability and colony-forming ability, while decreasing apoptosis and differentiation. An oncogenic role for miR-1246 has also been documented in relapsed T-cell ALL [37]. Increased expression of miR-1246 was found in relapsed patients compared to those in remission, and transfection of a T lymphocyte cell line with a miR-1246 mimic significantly elevated cell proliferation rates, suggesting a potential association with disease relapse.

Similarly to miR-1246, the upregulation of miR-1290 expression has been associated with the initiation and progression of ALL. Assays conducted in B-cell acute lymphoblastic leukemia (B-ALL) cell lines revealed increased levels of miR-1290 compared to control cells [38]. These findings were corroborated in pediatric patients with relapsed ALL, where significantly higher expression of miR-1290 was observed in comparison to healthy subjects [39]. Furthermore, the upregulation of miR-1290 correlates with poor relapse-free survival in pediatric ALL patients, with a survival rate of only 62% among those with miR-1290 levels exceeding the third quartile [40].

In relation to miR-223-3p, previous studies indicate that elevated levels of this microRNA enhance the survival, mobility, and invasion of T-cell acute lymphoblastic leukemia (T-ALL) cells [41]. Similar upregulation of miR-223-3p has been noted in T-cell prolymphocytic leukemia (T-PLL), where it is linked to alterations in DNA damage response and disruption of cell cycle regulators [42]. Additionally, increased expression of miR-223-3p is associated with reduced survival in T-PLL, with patients exhibiting lower miR-223-3p expression surviving a median of 26.0 months compared to 14.9 months for those with higher expression. Conversely, downregulation of miR-223-3p has been documented in AML patients relative to healthy individuals, with reduced expression linked to unfavorable cytogenetic groups, the presence of >50% blasts at diagnosis, and lower survival rates [43].

Despite prior investigations into the roles of miR-1290, miR-223-3p, and miR-1246 in leukemia development, their potential for predicting diagnosis and MRD status remains unexamined. Notably, individual miRNAs such as miR-367, miR-217, miR-107, miR-16-2-3p, miR-326, miR-335-3p, miR-125b-1, miR-203, miR-30, miR-143, miR-137, miR-101, miR-31, miR-32, miR-132, miR-129, and miR-124, as well as panels comprising multiple sequences, have demonstrated AUC values equal to or exceeding 0.80 for diagnosing ALL [13–17, 19], [44–46]. Notably, miR-24, miR-132, and miR-129 exhibited the highest AUC values (>0.95) and were found to be downregulated in the bone marrow of ALL patients compared to healthy controls [46]. Other miRNAs demonstrating excellent discriminatory ability (AUC ≥ 0.90) included miR-32, miR-31, miR-101, miR-143, miR-137, and miR-30, which were aberrantly expressed in plasma, whole blood, and bone marrow samples [19, 45, 46]. Among these, only miR-32 showed higher expression levels in the ALL group, while the remaining miRNAs exhibited lower levels compared to controls.

MiRNAs have also shown promise in identifying MRD status. Three miRNAs—miR-128-3p, miR-335-3p, and miR-326—demonstrated AUC values ranging from 0.80–0.97 [13, 16, 18, 20]. Among these, miR-128-3p exhibited the highest AUC value in distinguishing ALL patients with MRD greater than 1% from those with MRD less than 1% on day 15 of induction chemotherapy [20]. Plasma levels of miR-128-3p significantly decreased on days 15 and 33 of chemotherapy compared to the day of diagnosis (diagnosis-day 15: log_2_ FC ═ –4.49 and diagnosis-day 33: –2.23). In contrast, miR-335-3p and miR-326 effectively distinguished between MRD-positive and MRD-negative patients [13, 16]. Both miRNAs exhibited reduced expression in bone marrow samples from MRD-positive groups compared to MRD-negative groups one year after treatment, with AUC values of 0.80 and 0.84, respectively.

The AUC values reported in previous studies were higher than those observed in our study for the miRNAs with the greatest discriminatory capacity. Additionally, none of the miRNAs identified as potential MRD biomarkers (miR-128-3p, miR-335-3p, and miR-326) were detected in our population, likely due to differences in the biological samples utilized.

Prior studies have demonstrated that miRNA expression profiles are strongly sample-dependent. For instance, patients with esophageal squamous cell carcinoma (ESCC) exhibited significantly higher levels of miR-1246 in their serum and urine compared to healthy controls, yet no significant difference was observed in saliva [47]. Unlike blood, the production of saliva and urine can fluctuate significantly throughout the day, influenced by factors such as age, gender, circadian rhythm, diet, medication, and environmental exposure [47]. These factors can affect the expression levels of miRNAs in these fluids. Furthermore, technical aspects such as the RNA stabilizer used, the method of sample collection, and the bioinformatics alignment utilized can also influence salivary miRNA levels. We hypothesize that these factors may account for the discrepancies in dysregulated miRNAs identified in the saliva and urine of ALL patients in this study compared to blood-derived and bone marrow samples [28].

An additional consideration relevant to the interpretation of our findings is the discrepancy in the expression patterns of miR-1246 and miR-1290 between the discovery phase (upregulation) and the clinical validation phase (downregulation). This difference may stem from the distinct clinical and genetic profiles of the patient cohorts analyzed in each phase. Nonetheless, despite the heterogeneity between cohorts, miR-223-3p consistently exhibited an upregulated pattern in both phases, reinforcing its potential as a biomarker for MRD in pediatric ALL. In line with our results, Kyriakidis et al. (2025) recently identified miR-223-3p as a key biomarker for high-risk stratification and the differentiation of chemoresistant MRD-positive cases in childhood ALL using an automated machine learning approach. While miR-223-3p emerges as the most robust candidate, miR-1290 and miR-1246 should not be excluded but rather regarded as potential biomarkers warranting further investigation in future studies.

Despite our promising findings, this study has limitations, including the relatively small sample size used in both the discovery and validation phases. Further validation in a larger and more heterogeneous patient population is essential to ensure generalizability. Refinements in sample collection and preprocessing protocols are also necessary to minimize variability in miRNA expression, including standardization of oral hygiene procedures for saliva and staged centrifugation, hemolysis assessment, and creatinine normalization for urine samples. Additionally, optimizing miRNA isolation procedures from urine samples is crucial to ensure comprehensive representation of the miRNA population. These improvements could contribute to obtaining robust initial findings that can be subsequently validated using RT-qPCR.

Regarding the reference miRNAs used for normalization (miR-16-5p and miR-25), although geNorm identified them as the most stable candidates, their M-values (1.88) indicated considerable variability. Additional reference miRNAs could not be included, as the three candidates initially identified through sequencing failed to amplify. This limitation may have affected the robustness of the results. Future studies should consider alternative normalization strategies, such as spike-in controls or global mean approaches.

Moreover, the performance of the regression models used for ROC curve analyses may have been influenced by sociodemographic and clinical variables beyond MRD. Thus, future studies involving larger cohorts should incorporate these factors to provide more robust evidence regarding the diagnostic, prognostic, and predictive value of microRNAs.

Lastly, further research should assess the diagnostic value of the validated miRNAs within the pediatric ALL population to alleviate the challenges associated with bone marrow collection during diagnosis. Additionally, we strongly recommend evaluating the miRNAs’ ability to determine MRD status at other critical time points during chemotherapy and their association with patient outcomes. In our study, clinical data were collected up to day 15, and miRNA levels were measured exclusively at this time point; therefore, comparisons with day 33 miRNA levels or associations with clinical outcomes were not feasible.

## Conclusion

The results of this study suggest that miR-223-3p, either alone or in combination with miR-1290 and miR-1246, may serve as potential non-invasive biomarkers for MRD on day 15 in pediatric ALL. Rather than serving as a replacement for current bone marrow-based MRD monitoring, these findings should be interpreted as proof-of-concept adjuncts that highlight the promise of liquid biopsy approaches. This innovative strategy could significantly enhance pediatric patient care by alleviating the anxiety and discomfort associated with invasive procedures. However, further investigation in larger, multi-center cohorts is necessary to validate their clinical utility and potential integration into standard diagnostic and monitoring protocols.

## Acknowledgements

The authors express their gratitude to Dr. Sneider Gutierrez for his invaluable guidance in the bioinformatics analysis of the small RNA sequencing data. They also thank Dr. Robert Gilman for his partial funding of the sequencing analysis.

Furthermore, the authors extend their appreciation to the patients, their families, and the technical staff involved in the sample collection.

This manuscript was submitted as a pre-print in the link https://www.authorea.com/doi/full/10.22541/au.173225523.34040433/v1 [48].

## Supplemental data

Supplemental data are available at the following link: https://www.bjbms.org/ojs/index.php/bjbms/article/view/13119/4137.

## Data Availability

The data supporting this study’s findings are available from the corresponding author upon reasonable request.
